# Survival After Out-of-Hospital Cardiac Arrest Before and After Legislation for Bystander CPR

**DOI:** 10.1001/jamanetworkopen.2024.7909

**Published:** 2024-04-26

**Authors:** Siwen Li, Chongzhen Qin, Hongjuan Zhang, Mailikezhati Maimaitiming, Junyi Shi, YiKai Feng, Kepei Huang, Yanxin Bi, Minmin Wang, Qiang Zhou, Yinzi Jin, Zhi-Jie Zheng

**Affiliations:** 1 Shenzhen Center for Prehospital Care, Futian District, Shenzhen, China; 2 Department of Global Health, School of Public Health, Peking University, Beijing, China; 3 Institute for Global Health and Development, Peking University, Beijing, China

## Abstract

**Importance:**

The lack of evidence-based implementation strategies is a major contributor to increasing mortality due to out-of-hospital cardiac arrest (OHCA) in developing countries with limited resources.

**Objective:**

To evaluate whether the implementation of legislation is associated with increased bystander cardiopulmonary resuscitation (CPR) and automated external defibrillator (AED) use and improved clinical outcomes for patients experiencing OHCA and to provide policy implications for low-income and middle-income settings.

**Design, Setting, and Participants:**

This observational cohort study analyzed a prospective city registry of patients with bystander-witnessed OHCA between January 1, 2010, and December 31, 2022. The Emergency Medical Aid Act was implemented in Shenzhen, China, on October 1, 2018. An interrupted time-series analysis was used to assess changes in outcomes before and after the law. Data analysis was performed from May to October 2023.

**Exposure:**

The Emergency Medical Aid Act stipulated the use of AEDs and CPR training for the public and provided clear legal guidance for OHCA rescuing.

**Main Outcomes and Measures:**

The primary outcomes were rates of bystander-initiated CPR and use of AEDs. Secondary outcomes were rates of prehospital return of spontaneous circulation (ROSC), survival to arrival at the hospital, and survival at discharge.

**Results:**

A total of 13 751 patients with OHCA (median [IQR] age, 59 [43-76] years; 10 011 men [72.83%]) were included, with 7858 OHCAs occurring during the prelegislation period (January 1, 2010, to September 30, 2018) and 5893 OHCAs occurring during the postlegislation period (October 1, 2018, to December 31, 2022). The rates of bystander-initiated CPR (320 patients [4.10%] vs 1103 patients [18.73%]) and AED use (214 patients [4.12%] vs 182 patients [5.29%]) increased significantly after legislation implementation vs rates before the legislation. Rates of prehospital ROSC (72 patients [0.92%] vs 425 patients [7.21%]), survival to arrival at the hospital (68 patients [0.87%] vs 321 patients [5.45%]), and survival at discharge (44 patients [0.56%] vs 165 patients [2.80%]) were significantly increased during the postlegislation period. Interrupted time-series models demonstrated a significant slope change in the rates of all outcomes.

**Conclusions and Relevance:**

These findings suggest that implementation of the Emergency Medical Aid Act in China was associated with increased rates of CPR and public AED use and improved survival of patients with OHCA. The use of a systemwide approach to enact resuscitation initiatives and provide legal support may reduce the burden of OHCA in low-income and middle-income settings.

## Key Points

**Question:**

Is the implementation of legislation associated with increased bystander cardiopulmonary resuscitation (CPR) and automated external defibrillator (AED) use and improved clinical outcomes for patients experiencing out-of-hospital cardiac arrest (OHCA) in low-resource countries?

**Findings:**

In a cohort study of 13 751 patients with OHCA in Shenzhen, China, the pilot city for the CPR legislation, the rates of bystander-initiated CPR, AED use, prehospital return of spontaneous circulation, survival to arrival at the hospital, and survival at discharge were significantly increased during the postlegislation period. Interrupted time-series models demonstrated a significant slope change in the rates of all outcomes after legislation was implemented.

**Meaning:**

These findings suggest that the implementation of legislation is positively associated with bystander CPR and public defibrillation and improved survival among patients with OHCA.

## Teaser

This cohort study examines whether the implementation of legislation is associated with increased bystander cardiopulmonary resuscitation (CPR) and automated external defibrillator use and improved clinical outcomes for patients experiencing out-of-hospital cardiac arrest in China.

## Introduction

Out-of-hospital cardiac arrest (OHCA) is a major public health issue worldwide. Although resuscitation strategies have witnessed substantial evolution, survival rates from OHCA remain lower than 10% in most of the world. The global burden of OHCA is also a manifestation of inequality in that nearly 10% of approximately 350 000 incident cases survive in the US,^1^ and only 1.2% of approximately 1 340 000 cases survive in China annually.^2^ The survival rate significantly increases with early cardiopulmonary resuscitation (CPR) and defibrillation with bystander use of automated external defibrillators (AEDs).^3^ To increase rates of bystander CPR and AED use, many developed countries have implemented CPR training for the public and initiated public-access automated external defibrillation (PAD) programs.^4,5,6^ Although these efforts have yielded substantial progress toward improved survival following OHCA,^7,8^ governmental legislation can help by mandating CPR training or providing a disclaimer for bystander CPR and AED use, which is a key contributor to the effectiveness of an implementation strategy.^9,10^ Governmental legislation on bystander CPR and AED use has been enacted in many developed countries since 2000, aiming to provide legal protection for non–emergency medical services (EMS)–certified personnel using AEDs in an emergency situation and to regulate the public use of AED.^4,11,12,13^ However, the lack of evidence-based implementation strategies for bystander CPR and AED use is a major barrier for increasing survival after OHCA in developing countries with limited resources.^14^

China is among the first developing countries to adopt a systemwide approach for improving the survival of patients with OHCA in pilot cities, with an initiative of legislation to promote implementation strategies. At the start of 2010, the pilot cities implemented CPR training for the public and PAD programs, which involved the government, nongovernmental organizations, medical institutions, and communities in collaboration to perform basic life support skills training (including AED use skills) for the public, and encouraging social donations and self-purchasing to expand the deployment scale of AEDs. In 2018, the governments of pilot cities enacted the Emergency Medical Aid Act to promote implementation of the PAD programs. This law stipulated the deployment plan, management, use of AED, and basic life support skills training for the public and clarified the legal responsibility of prehospital dispatchers, bystander CPR, and AED use to provide clear legal guidance for improving emergency medical care for OHCA. The government provided unified standardized CPR training to medical workers, staff in subway stations, students, and the general public every year. By the end of 2022, a total of 200 000 individuals were trained such that approximately 3.7% of the overall population received emergency skills training in the pilot city. However, whether adopting governmental legislation in developed countries is applicable in developing countries with limited resources remains unknown.

To fill these research gaps, we conducted an interrupted time-series analysis based on individual-level data of the OHCA registry in a pilot city between 2010 and 2022. In the present study, we evaluated the association of governmental legislation regarding CPR training for the public and bystander CPR and AED use with survival from OHCA in China. These findings can provide policy implications for other developing countries with limited resources to reduce the OHCA burden.

## Methods

### Study Design and Participants

This observational cohort study was approved by the ethics committee of Peking University. According to China’s legislation, individual informed consent is not required for register-based studies. This study is reported following the Strengthening the Reporting of Observational Studies in Epidemiology (STROBE) reporting guidelines.

Shenzhen was the first pilot city to have a resuscitation strategy in China. Shenzhen has a population-based registry of OHCAs, the first and with the longest follow-up time, and the city has complete records of AED application and bystander information for patients with OHCA in China. This registry system includes all patients with OHCA, regardless of arrest causes, for whom resuscitation was attempted and who were then transported to a medical institution by EMS. Patients who achieved return of spontaneous circulation (ROSC) without bystander defibrillation before the arrival of EMS staff were excluded from the OHCA registry. EMS personnel completed the data form, which was confirmed by the physician in charge of patients and then systematically checked by the Shenzhen Emergency Medical Center. Data elements and definitions in this registry system were developed according to the Utstein template, including patients’ demographic characteristics, arrest on site, EMS dispatch, prehospital emergency medical intervention, treatment in the emergency department of the hospital, in-hospital clinical outcomes, follow-up, and bystander information (eTable 1 in Supplement 1).

We selected individuals after OHCA from this registry between January 1, 2010, and December 31, 2022. Individuals with missing important variables, without cardiac arrest witnesses, or younger than 18 years old were excluded in this study. We divided patients into 2 groups according to whether the time of OHCA occurred before (January 1, 2010, to September 30, 2018) or after (October 1, 2018, to December 31, 2022) the enactment of legislation (eFigure 1 in Supplement 1).

### Outcomes and Covariates

Primary outcomes of this study were rates of receiving bystander CPR and AED use in patients with OHCA. The rates of any prehospital ROSC, survival to arrival at the hospital (pulse at hospital arrival), and survival at discharge in patients with OHCA were secondary outcomes.

Covariates included patient age, sex, origin of arrest, arrest location, type of CPR, dispatcher instruction CPR, and time interval between collapse and EMS contact with patient. Age and sex were self-reported and extracted from the electronic medical records; these were included in the analysis given the past literature demonstrating that age and sex can be associated with the risk of receiving CPR provided by a bystander.^15^ The origin of arrest and location of arrest may be associated with AED use and clinical outcomes.^16^

### Statistical Analysis

Data analysis was performed from May to October 2023. Data from the 13-year study period were used to calculate the rates of EMS-assessed OHCA with bystander CPR and AED use. The rate of EMS-assessed OHCA with bystander CPR and/or AED use was calculated as the total number of EMS-assessed OHCAs with bystander CPR and/or AED use per 3 months divided by the total population served by EMS agency per 3 months. The total rates of EMS-assessed OHCA with bystander CPR and/or AED use in the prelegislation period and postlegislation period were calculated as the total number of EMS-assessed OHCAs with bystander CPR and/or AED use in each stratum divided by the total population served by the EMS agency in each stratum.

After inspecting the completeness and distribution of the data, we summarized descriptive statistics regarding the frequency and proportion of demographic characteristics and specified outcomes stratified by time before and after legislation implementation. Calculations excluded missing values. Univariable comparisons were made with likelihood-ratio χ^2^ tests for categorical variables and Wilcoxon rank-sum tests for continuous variables. Multivariable logistic regression analysis was used to assess the association of implemented legislation with outcomes. Odds ratio (ORs) and 95% CIs of each outcome for postlegislation period were calculated with adjustment potential confounders, including age, sex, origin of arrest, location of arrest, type of CPR, dispatcher instruction CPR, and time interval between collapse and EMS contact with the patient. An interrupted time-series analysis was conducted using generalized linear models based on the gaussian distribution with an identity link function to examine whether there was an immediate level change in outcomes before and after the implemented legislation, as well as whether there were changes in trends of the rate of all outcomes. In these models, the dependent variables were the outcome variables, and the explanatory variables were time (season), an indicator of legislation, and an interaction term representing the number of months after legislation implementation. Considering that there would be a lag effect of legislation implementation, we chose 1.5 years after implementation of the legislation as the time of intervention to assess the association of legislation with AED use. A 2-sided *P* < .05 was used to indicate statistical significance. All other analyses were conducted using Stata statistical software version 17 (StataCorp).

## Results

### Characteristics

A total of 13 751 individuals who experienced OHCA in front of witnesses were identified from January 1, 2010, to December 31, 2022, with 7858 OHCAs occurring during the prelegislation period and 5893 OHCAs occurring during the postlegislation period. Characteristics of the cohort are presented in Table 1. For the overall cohort, the median (IQR) age was 59 (43-76) years; 10 011 patients (72.83%) were male and 3740 patients (27.17%) were female. During the postlegislation period, the proportion of patients with OHCA who received bystander CPR was significantly increased (320 patients [4.10%] vs 1103 patients [18.73%]) vs the prelegislation period; the rate of dispatcher instruction during CPR (7 patients [2.19%] vs 118 patients [10.07%]) and AED use (214 patients [4.12%] vs 182 patients [5.29%]) also increased significantly. The prevalence of patients with OHCA for whom AED was administered by EMS was significantly decreased (211 patients [4.07%] vs 33 patients [0.96%]). The time between collapse and emergency call (mean [SD], 14.57 [5.65] minutes vs 14.01 [10.22] minutes) and the time between collapse and EMS contact with the patient (mean [SD], 26.92 [10.18] minutes vs 26.29 [10.25] minutes) both presented a small but statistically significant reduction after legislation implementation. The proportions of patients with OHCA with prehospital ROSC (72 patients [0.92%] vs 425 patients [7.21%]; increase of 6.29%), survival to arrival at the hospital (68 patients [0.87%] vs 321 patients [5.45%]; increase of 4.58%), and survival at discharge (44 patients [0.56%] vs 165 patients [2.80%]; increase of 2.24%) were higher after the implementation of legislation (Table 1).

**Table 1.** Characteristics of Patients With Bystander-Witnessed Out-of-Hospital Cardiac Arrest Overall and by Legislation Period

Characteristic	Patients, No. (%)			*P* value
	Total (N = 13 751)^a^	Prelegislation period (n = 7858)^b^	Postlegislation period (n = 5893)^c^	
Age, median (IQR), y	59 (43-76)	56 (41-74)	62 (47-80)	<.001
Sex				
Male	10 011 (72.83)	5761 (73.31)	4250 (72.19)	<.001
Female	3740 (27.17)	2097 (26.69)	1643 (27.81)	
Origin of arrest				
Cardiac	362 (2.66)	158 (2.05)	204 (3.47)	<.001
Presumed cardiac	921 (6.77)	493 (6.38)	428 (7.28)	
Traumatic	555 (4.08)	325 (4.21)	230 (3.91)	
Drown	13 (0.10)	5 (0.06)	8 (0.14)	
Suffocate	17 (0.12)	5 (0.06)	12 (0.20)	
Overdose or poisoning	62 (0.46)	39 (0.50)	23 (0.39)	
Electric shock	117 (0.86)	80 (1.04)	37 (0.63)	
Other	11 555 (84.95)	6618 (85.70)	4937 (84.00)	
Location of arrest				
Home	7719 (56.13)	4015 (51.09)	3704 (62.85)	<.001
Workplace	1420 (10.33)	574 (7.30)	846 (14.36)	
Public place	456 (3.32)	82 (1.04)	374 (6.35)	
Medical organization	73 (0.53)	15 (0.19)	58 (0.98)	
Road	989 (7.19)	599 (7.62)	390 (6.62)	
Ambulance	80 (0.58)	33 (0.42)	47 (0.80)	
Other	3014 (21.92)	2540 (32.32)	474 (8.04)	
Bystander CPR	1423 (10.37)	320 (4.10)	1103 (18.73)	<.001
Bystander who initiated CPR				
Family member	505 (35.41)	134 (41.61)	371 (33.61)	<.001
Patient companion^d^	242 (16.97)	40 (12.42)	202 (18.30)	
On-site staff^e^	191 (13.39)	13 (4.04)	178 (16.12)	
Health care workers^f^	205 (14.38)	86 (26.71)	119 (10.78)	
Passers-by	99 (6.94)	13 (4.04)	86 (7.79)	
EMS	77 (5.61)	31 (10.25)	46 (4.26)	
Other	104 (7.29)	3 (0.93)	101 (9.15)	
Type of bystander CPR				
Chest compression–only CPR	859 (60.37)	182 (56.87)	677 (61.38)	<.001
Conventional CPR with rescue breathing	498 (35.00)	109 (34.06)	389 (35.27)	
Only rescue breathing	10 (0.70)	7 (2.19)	3 (0.27)	
Advanced CPR^g^	56 (3.94)	22 (6.88)	34 (3.08)	
Receipt of dispatcher instruction during CPR	125 (8.78)	7 (2.19)	118 (10.70)	<.001
Automated external defibrillator used	396 (4.59)	214 (4.12)	182 (5.29)	<.001
Initial public access defibrillation				
Family member	5 (0.06)	0	5 (0.15)	<.001
Patient companion^d^	7 (0.08)	0	7 (0.20)	
On-site staff^e^	69 (0.80)	3 (0.06)	66 (1.92)	
Health care workers^f^	55 (0.64)	0	55 (1.60)	
Passers-by	14 (0.16)	0	14 (0.41)	
EMS	244 (2.83)	211 (4.07)	33 (0.96)	
Other	2 (0.02)	0	2 (0.18)	
Time between collapse and emergency call, mean (SD), min	14.39 (7.94)	14.57 (5.65)	14.01 (10.22)	<.001
Time between collapse and EMS contact with patient, mean (SD), min	26.65 (10.22)	26.92 (10.18)	26.29 (10.25)	.004
Time between collapse and first shock, mean (SD), min	11.49 (10.14)	12.45 (10.05)	11.17 (10.83)	
Time between collapse and hospital arrival, mean (SD), min	48.99 (26.14)	49.51 (27.94)	48.38 (23.84)	
Prehospital return of spontaneous circulation	497 (3.61)	72 (0.92)	425 (7.21)	<.001
Survival to arrive at hospital	389 (2.83)	68 (0.87)	321 (5.45)	<.001
Survival at discharge	209 (2.11)	44 (0.56)	165 (2.80)	<.001

Abbreviations: CPR, cardiopulmonary resuscitation; EMS, emergency medical services.

^a^ Refers to the entire period of January 1, 2010, to December 31, 2022.

^b^ Refers to January 1, 2010, to September 30, 2018.

^c^ Refers to October 1, 2018, to December 31, 2022.

^d^ Refers to friends, colleagues, classmates, or life partners.

^e^ Refers to the individuals who worked in the places where arrest occurred (eg, shopping center, sports center, and traffic stations).

^f^ Refers to individuals who work in health care center or nursing house.

^g^ Advanced CPR means application of assistive devices and medications on top of basic life support to establish more effective ventilation and circulation, as well as continuously monitor electrocardiogram, blood pressure, pulse rate, and oxygen saturation.

After controlling for confounding factors, the risk of not receiving bystander CPR (OR, 0.20; 95% CI, 0.11-0.29; *P* < .001) and the risk of not receiving defibrillation by AED (OR, 0.01; 95% CI, 0.01-0.09; *P* < .001) were lower in the postlegislation period vs the prelegislation period (Table 2). Compared with the prelegislation period, patients with OHCA had lower risk of unfavorable clinical outcomes in the postlegislation period, including non–prehospital ROSC (OR, 0.19; 95% CI, 0.14-0.25; *P* < .001), nonsurvival to arrival at the hospital (OR, 0.82; 95%, 0.71-0.96; *P* < .001), and nonsurvival at discharge (OR, 0.07; 95% CI, 0.01-0.32; *P* < .001) (Table 2 and eFigure 2 in Supplement 1).

**Table 2.** Association of Legislation Implementation With Outcomes, Controlling for Baseline in Multivariable Logistic Regression^a^

Intervention period	Bystander CPR		AED defibrillation		Prehospital ROSC		Survival to arrive at hospital		Survival at discharge	
	OR (95% CI)	*P* value	OR (95% CI)	*P* value	OR (95% CI)	*P* value	OR (95% CI)	*P* value	OR (95% CI)	*P* value
Pre legislation	1 [Reference]	NA	1 [Reference]	NA	1 [Reference]	NA	1 [Reference]	NA	1 [Reference]	NA
Postlegislation	0.20 (0.11-0.29)	<.001	0.01 (0.01-0.09)	<.001	0.19 (0.14-0.25)	<.001	0.82 (0.71-0.96)	<.001	0.07 (0.01-0.32)	<.001

Abbreviations: AED, automated external defibrillator; CPR, cardiopulmonary resuscitation; NA, not applicable; OR, odds ratio; ROSC, spontaneous circulation.

^a^ Adjusted for age, sex, origin of arrest, location of arrest, type of CPR, dispatcher instruction CPR, and time interval between collapse and emergency medical services contact with the patient. The outcome events were defined as not receiving CPR, not receiving defibrillation by AED, non–prehospital ROSC, nonsurvival to arrive at hospital, and nonsurvival at discharge in the models.

**Figure 1.**
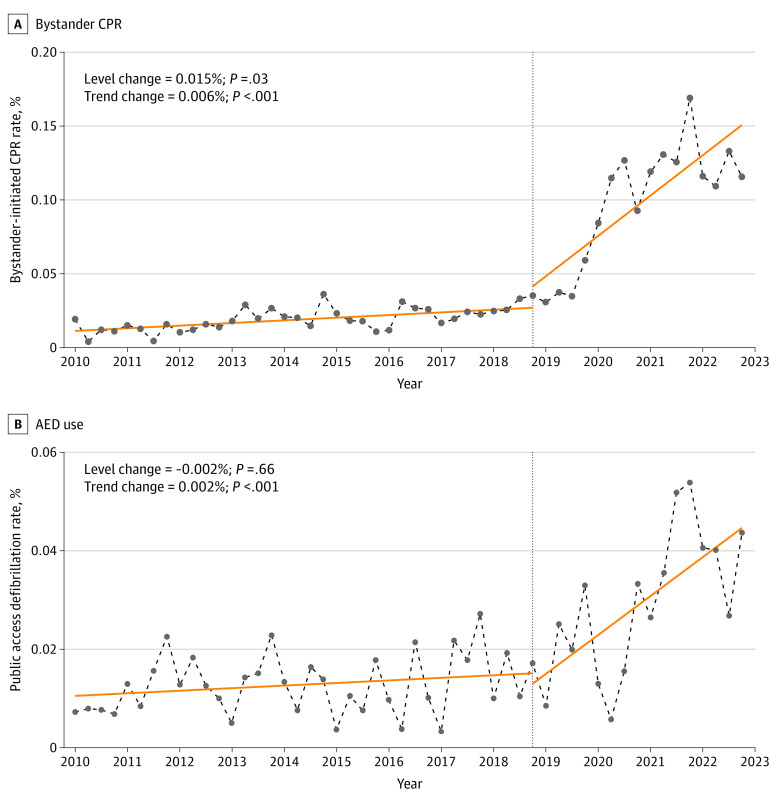
Rates and Estimates of Bystander Cardiopulmonary Resuscitation (CPR) and Automated External Defibrillator (AED) Use in Interrupted Time-Series Analysis Solid lines denote the estimated trend of the outcomes. Circles and dashed lines denote the real trend of the outcomes. Vertical dashed line denotes the time of intervention implementation.

Table 3 shows that the rate of bystander CPR immediately increased by 0.015% at the time of legislation implementation. After legislation implementation, this rate began increasing by 0.004% per season, representing a small but statistically significant change in bystander CPR trend (trend change, 0.006% per season; 95% CI, 0.004%-0.010%). At the point the legislation was implemented, the rate of defibrillation by AED immediately decreased by approximately 0.002%, but the trend in the rate of defibrillation by AED changed significantly (trend change, 0.002% per season; 95% CI, 0.001%-0.003%) (Figure 1). At 1.5 years after the legislation was implemented, there was a positive step change in the level of AED use, with the rate beginning to increase by 0.001% per season at this point, which indicates that the trend in the rate of defibrillation by AED changed significantly (trend change, 0.003% per season; 95% CI, 0.001%-0.005%) (eTable 2 and eFigure 3 in Supplement 1).

**Table 3.** Changes in Outcomes Before and After Implementation of Legislation in Multivariable Gaussian Regression

Outcome	Level change, % (95% CI)	*P* value	Trend change, % (95% CI)	*P* value
Bystander cardiopulmonary resuscitation	0.015 (0.010 to 0.039)	.03	0.006 (0.004 to 0.010)	<.001
Public access defibrillation	−0.002 (−0.011 to 0.007)	.66	0.002 (0.001 to 0.003)	<.001
Prehospital return of spontaneous circulation	0.014 (0.003 to 0.026)	.01	0.004 (0.002 to 0.005)	<.001
Survival to arrive at the hospital	0.017 (0.01 to 0.03)	.05	0.002 (0.001 to 0.004)	.04
Survival at discharge	0.008 (0.001 to 0.014)	.02	0.001 (−0.001 to 0.002)	.07

**Figure 2.**
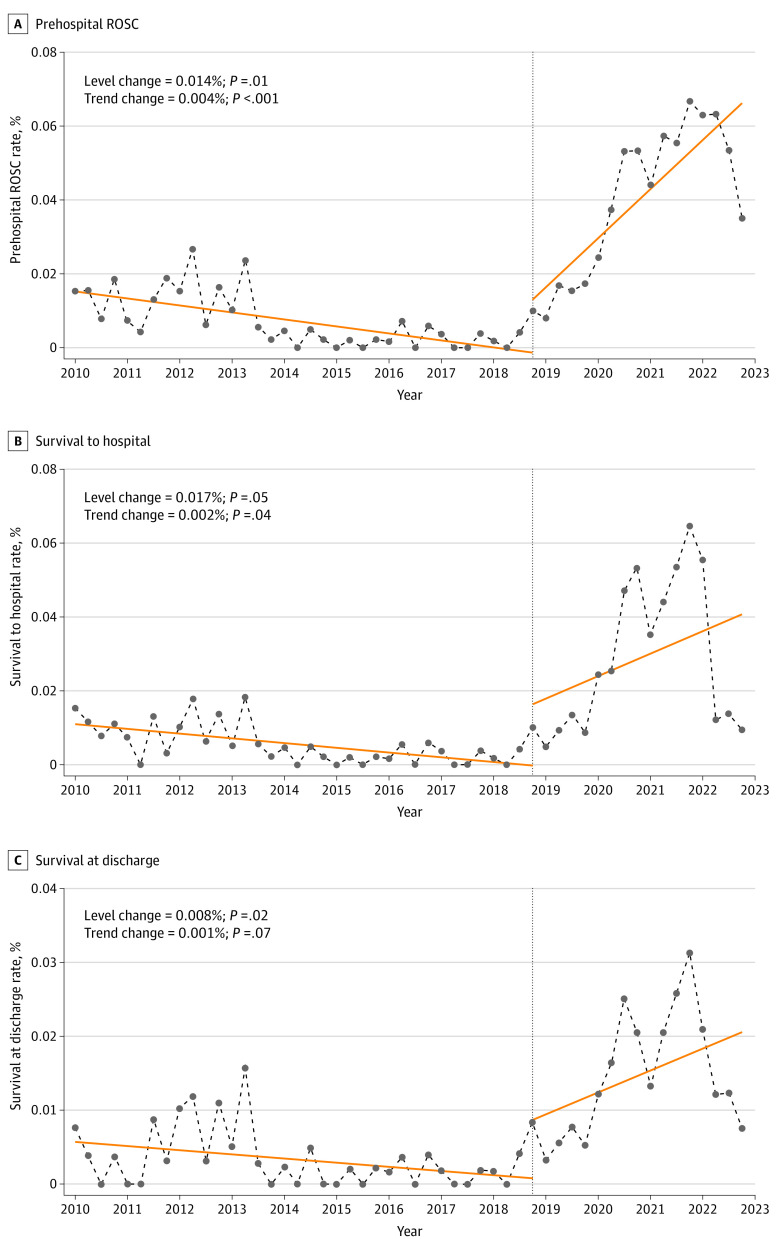
Rates and Estimates of Prehospital Return of Spontaneous Circulation (ROSC), Survival to Arrival at the Hospital, and Survival at Discharge in Interrupted Time-Series Analysis Solid lines denote the estimated trend of the outcomes. Circles and dashed lines denote the real trend of the outcomes. Vertical dashed line denotes the time of intervention implementation.

The rates of prehospital ROSC and survival to arrival at the hospital were both immediately increased by 0.014% and 0.017%, respectively (Table 3). After legislation implementation, the rates of prehospital ROSC and survival to arrival at the hospital both began to increase by approximately 0.004% per season, presenting a significant change in trends of the prehospital ROSC rate (trend change, 0.004% per season; 95% CI, 0.002%-0.005%) and survival to arrival at the hospital (trend change, 0.002% per season; 95% CI, 0.001%-0.004%). No significant reduction in the rate of survival at discharge was noted (Table 3 and Figure 2).

## Discussion

To our knowledge, this cohort study is the first to investigate the association of governmental legislation for CPR training for the public, bystander CPR, and AED use with survival from OHCA in developing countries using interrupted time-series analysis. The present study provides evidence that the implementation of legislation will likely improve survival after OHCA by increasing rates of bystander CPR and AED use. The systemwide approach for improving the survival of OHCA in pilot cities of China with an initiative of legislation can offer other developing countries a tool that can be tailored to meet local needs while contributing to promoting the implementation of resuscitation strategies.

In this study, we found that implementing governmental legislation was associated with statistically significant increases in the rates of receiving bystander CPR and defibrillation using AED for patients after OHCA. Furthermore, we identified not only an immediate increase in the rates of bystander CPR and AED use but persistent trends of increase in both rates during the months following implementation. These observations suggest that the interventions were practical and sustainable. Our findings were consistent with previous studies^17^ evaluating the association of legislation with bystander CPR outcomes in the US, reporting that the rates of bystander CPR and bystander AED in states with associated laws were higher than those in states without such laws, including those requiring CPR instruction in high schools. This may be explained by the fact that the enactment of legislation has a positive effect on broadening CPR training for the public and increasing CPR and AED administration.^18,19,20,21^ In low-resource settings, the government can adopt a systemwide approach to implement resuscitation strategies and identify and correct the inefficiency caused by unclear division of responsibilities among health system departments.^22^ Legislation can provide legal support for this strategy by regulating the identification of clinicians and modes of CPR training for the public, as well as clarifying the responsibilities of health system departments. This is very important for countries with limited resources because it can reduce the problem of increased CPR training costs caused by unclear division of responsibilities among health system departments. The Emergency Medical Aid Act provides clear resource allocation and establishes AED priority deployment plans, which may be another reason for the increased rates of bystander AED use.^23,24^ A systemwide approach advocates for low-resource countries to implement health strategies from a holistic perspective of the health system to determine priority health care services. The enactment of legislation can play a guiding role in the implementation of resuscitation strategies. This approach can help low-income and middle-income countries reasonably allocate resources to promote the development and efficient deployment of resuscitation strategies.

In addition to identifying an increase in the rates of bystander CPR and use of AED defibrillation, we found that rates of prehospital ROSC increased by 6.29%, rates of survival to arrival at the hospital increased by 4.58%, and rates of survival at discharge increased by 2.24% in the postlegislation period. We also found that the effectiveness of legislation was evident and showed a long-term trend. These findings suggest that legislation implementation may be associated with improved clinical outcomes of patients after OHCA. Similar findings have been observed in other studies.^25,26^ Compared with countries without related laws, those with such laws have better management of OHCA, which can increase the patient survival rate and improve neurological outcomes.^27^ These findings may be attributed to implementation of the Emergency Medical Aid Act and may strengthen governmental management of the emergency medical system, which is beneficial for improving the response time and clinical outcomes of individuals after OHCA.^28,29^ In the process of developing a resuscitation strategy using a systemwide approach, governments should strengthen its management by improving accountability. The formulation of legislation provides a legal basis and guarantee of accountability. EMS is an important part of improving survival outcomes in patients with OHCA. The Chinese government formulates laws and regulations governing the service processes and standards of the emergency medical system, ensuring that all aspects of EMS behavior are regulated and constrained by law, thereby improving the efficiency and quality of resuscitation strategy implementation.^30,31^ The implementation of health strategies in low-income countries through this joint approach is of great importance because it can help to improve the alignment of health plans with the ultimate goals.

### Limitations

Our study has some limitations. First, this was an observational study; thus, it has inherent risks of bias that cannot be controlled. Second, because the registry system of OHCAs of Shenzhen does not gather data on the neurological trajectory, this study could not assess the neurological outcomes. Third, we did not have variables on bystanders for which we could control as confounders in the multivariable logistic regression analysis. Fourth, this study cannot control for unrecorded variables, such as the quality of bystander CPR, which may cause bias in the results. Poor CPR quality may decrease survival in patients with OHCA.^32^ Such information is essential for a more precise evaluation of the effectiveness of legislation. Therefore, to examine these factors, future studies are warranted.

## Conclusion

Our findings suggest that the implementation of legislation could effectively increase the rates of patients with OHCA receiving bystander CPR and AED use and contribute to improving prehospital ROSC and survival at discharge. Our findings suggest that using a systemwide approach to enact resuscitation initiatives and providing supportive laws may help promote the implementation of resuscitation strategies in developing countries to reduce the burden of OHCA.

## Notes

Supplement 1. **eFigure 1.** Study Flowchart **eFigure 2.** The Associations Between Legislation and Bystander CPR, AED Defibrillation, and Clinical Outcomes of OHCAs **eFigure 3.** Rates and Estimates of AED Using the 1.5 Years After Implementation of Legislation in Interrupted Time-Series Analysis **eTable 1.** Data Elements in the Registry System **eTable 2.** Changes in AED Use in the 1.5 Years After Implementation of Legislation in Multivariable Gaussian Regression

Supplement 2.
Data Sharing Statement
