# Supplementary material for: Survival After Out-of-Hospital Cardiac Arrest Before and After Legislation for Bystander CPR
Source: JAMA Netw Open. 2024 Apr 26;7(4):e247909. doi: 10.1001/jamanetworkopen.2024.7909 (PMC11053379; doi:10.1001/jamanetworkopen.2024.7909)
Supplement: Supplement 1. — eFigure 1. Study Flowchart eFigure 2. The Associations Between Legislation and Bystander CPR, AED Defibrillation, and Clinical Outcomes of OHCAs eFigure 3. Rates and Estimates of AED Using the 1.5 Years After Implementation of Legislation in Interrupted Time-Series Analysis eTable 1. Data Elements in the Registry System eTable 2. Changes in AED Use in the 1.5 Years After Implementation of Legislation in Multivariable Gaussian Regression [file jamanetwopen-e247909-s001.pdf]

Supplemental Online Content

Li S, Qin C, Zhang H, et al. Survival after out-of-hospital cardiac arrest before and after legislation for bystander CPR. *JAMA Netw Open*. 2024;7(4):e247909. doi:10.1001/jamanetworkopen.2024.7909

eFigure 1. Study Flowchart
eFigure 2. The Associations Between Legislation and Bystander CPR, AED Defibrillation, and Clinical Outcomes of OHCAs
eFigure 3. Rates and Estimates of AED Using the 1.5 Years After Implementation of Legislation in Interrupted Time-Series Analysis
eTable 1. Data Elements in the Registry System
eTable 2 Changes in AED Use in the 1.5 Years After Implementation of Legislation in Multivariable Gaussian Regression

This supplemental material has been provided by the authors to give readers additional information about their work.

© 2024 Li S et al. *JAMA Network Open.*

eFigure 1. Study Flowchart

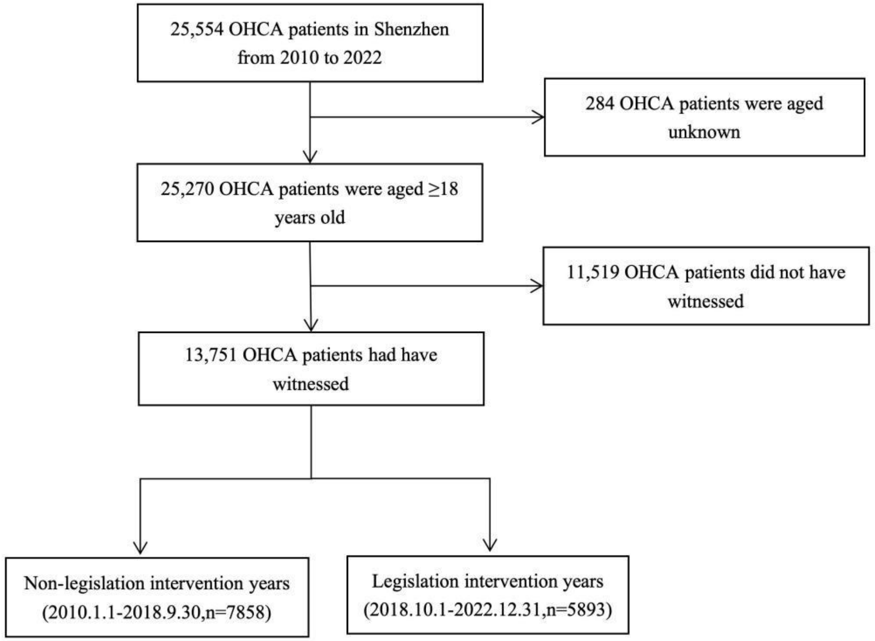

© 2024 Li S et al. *JAMA Network Open.*

eFigure 2. The Associations Between Legislation and Bystander CPR, AED Defibrillation, and Clinical Outcomes of OHCAs

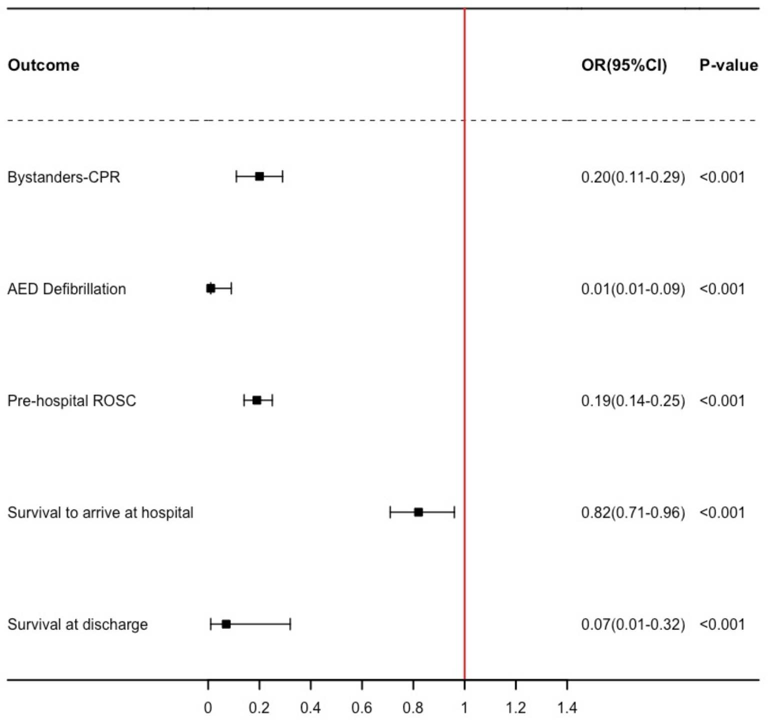

The outcome events of this logistic regression model were defined as the non-bystander CPR, non-AED use, no survival to arrive at hospital(death arrive at hospital), no survival at discharge(death at discharge). OR less than of 1 indicated the legislation is a protective factor against the occurrence of outcome event.

© 2024 Li S et al. *JAMA Network Open.*

eFigure 3. Rates and Estimates of AED Using the 1.5 Years After Implementation of Legislation in Interrupted Time-Series Analysis

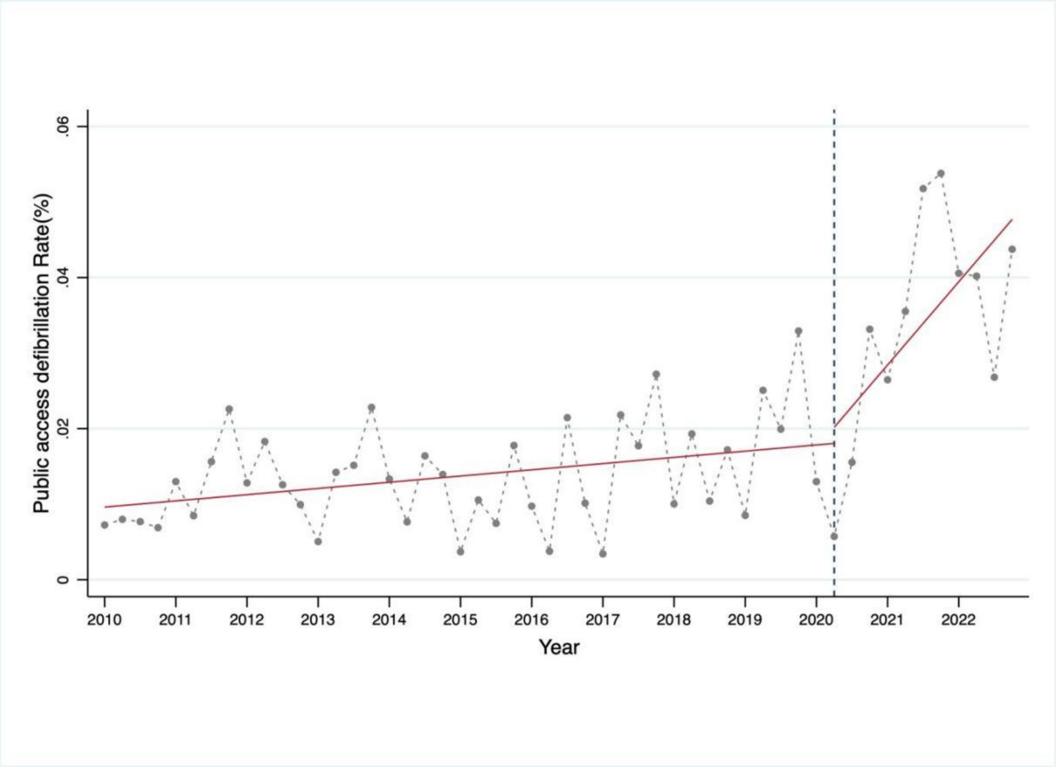

© 2024 Li S et al. *JAMA Network Open.*

eTable 1. Data Elements in the Registry System

Elements	Variables	Definition
Demographic characteristics	Age	
	Gender	1= male;2=female;3=other
	Race	1=Han;2=other
	Hypertension	1=Yes;2=No
	Diabetes	1=Yes;2=No
	Coronary heart disease	1=Yes;2=No
	Heart failure	1=Yes;2=No
	Stroke	1=Yes;2=No
	Cancer	1=Yes;2=No
	Smoking	1=Yes;2=No
	drinking	1=Yes;2=No
Arrest information	Arrest time	
	Location	1=Home; 2=Work place; 3=Public place;4=Medical organization; 5=Road;6=Ambulance;7=Other
	Cause	1=Cardiac;2= Non-cardiac
	Witness type	1=Families;2=Patients patterns;3=Staff on; 4=Medical staff;5=Pass by;6=EMS;7=Other;8=None
	Witness time	
	Bystander CPR	1=Yes;2=No
	Bystander CPR type	1=Chest-compression-only CPR; 2=Conventional CPR with rescue breathing; 3=Only rescue breathing;4=Advanced CPR
	Identity of bystander-initiated CPR	1=Families;2=Patients patterns;3=Staff on;4=Medical staff;5=Pass by;6=EMS;7=other

© 2024 Li S et al. *JAMA Network Open.*

	Apply AED	1=Yes;2=No
	Identify of apply AED	1=Families;2=Patients patterns;3=Staff on;4=Medical staff;5=Pass by;6=EMS; 7=Othe
	bystander CPR guided by dispatcher	1=Yes;2=No
	Call EMS time	
	Ambulance dispatch time	
	Ambulance arrival time at the scene	
	Ambulance departure time from the scene	
	First CPR time	
	First defibrillation time	
	First monitoring of heart rhythm	
	Pre-ROSC	1=Yes;2=No
	Outcome at the scene	1=transport to the hospital; 0=Death
Pre-hospital Emergency medical intervention information	Infusion pathway	1=Venous pathway; 2=Other pathway; 3=No pathway
	Successfully established advanced airway before arrival at hospital	1=Yes;2=No
	Drug use	1=Yes;2=No
Emergency department information	Ambulance arrival time at the emergency department	
	Patient's status upon arrival at the emergency department	
	24-hour emergency outcome	1=Stop resuscitation;2=Admitted to the hospital; 3=Transfer to other hospital;4=Give up treatment
in-hospital information	Survival at discharge	1=Yes;2=No

© 2024 Li S et al. *JAMA Network Open.*

	Time at discharge	
	CPC score at discharge	1=CPC1;2=CPC2;3=CPC3;4=CPC=4;5=CPC5

© 2024 Li S et al. *JAMA Network Open.*

eTable 2 Changes in AED Use in the 1.5 Years After Implementation of Legislation in Multivariable Gaussian Regression

**Outcomes**	**Level change(95%CI)**	**P-value**	**Trend change(95%CI)**	***P-value***
Public access defibrillation	0.002(-0.012,0.016)	*.762*	0.003(0.001,0.005)	.039

© 2024 Li S et al. *JAMA Network Open.*
