# Supplementary material for: Survival After Out-of-Hospital Cardiac Arrest Before and After Legislation for Bystander CPR
Source: JAMA Netw Open. 2024 Apr 26;7(4):e247909. doi: 10.1001/jamanetworkopen.2024.7909 (PMC11053379; doi:10.1001/jamanetworkopen.2024.7909)
Supplement: Supplement 2. — Data Sharing Statement [file jamanetwopen-e247909-s002.pdf]

Data Sharing Statement

Li. Survival After Out-of-Hospital Cardiac Arrest Before and After Legislation for Bystander CPR. *JAMA Netw Open*. Published April 26, 2024. doi:10.1001/jamanetworkopen.2024.7909

Data
Data available: No
